# Assessing the predictive validity of the UCAT—A systematic review and narrative synthesis

**DOI:** 10.1080/0142159X.2021.1998401

**Published:** 2021-11-23

**Authors:** Laksha Bala, Stephen Pedder, Amir H. Sam, Celia Brown

**Affiliations:** a Imperial College School of Medicine, Imperial College London, London, UK; b Warwick Medical School, University of Warwick, Coventry, UK

**Keywords:** Assessment, medicine, predictive, validity, UCAT

## Abstract

**Background:**

The University Clinical Aptitude Test (UCAT) is an admissions assessment used by a consortium of universities across the UK, Australia, and New Zealand, to aid the selection of applicants to medical and dental degree programmes. The UCAT aims to measure the mental aptitude and professional behaviours required to become successful doctors and dentists. We conducted a systematic review to establish the predictive value of the UCAT across measures of performance at undergraduate and post-graduate levels.

**Methods:**

A literature search was conducted in April 2020 using eight electronic databases: MEDLINE, APA PsycInfo, SCOPUS, Web of Science, EThOS, OpenGrey, PROSPERO, and the UCAT website. Data were extracted from selected studies and tabulated as results matrices. A narrative synthesis was performed.

**Results:**

Twenty-four studies satisfied our inclusion criteria, 23 of which were deemed to be of good quality (using the Newcastle-Ottawa Scale). For over 70% of univariate data points, the UCAT exerted no statistically significant predictive validity; for the remainder, predictive power was weak. The cognitive total and verbal reasoning subtests had the largest evidence base as weakly positive predictors of academic performance. The SJT subtest was a weak predictor of professional behaviour during medical school. Studies specific to dental schools demonstrated variable findings across the five studies. Only 1 study looked at post-graduate outcome measures and demonstrated that the UCAT was not a predictor of health- or conduct-related fitness to practice declarations at GMC registration.

**Conclusions:**

These data provide some support for the use of cognitive total and verbal reasoning subtests as part of medical school selection. Further research is needed to investigate outcomes beyond professional registration and for dental students.

## Introduction

The UCAT (introduced in 2006, and known until 2019 as the UKCAT) is an aptitude test deployed by a consortium of universities in the selection of medical and dental students for both undergraduate and graduate-entry programmes. It is one of several selection tools including aptitude tests, academic records, personal statements, references, situational judgment tests (SJTs), personality and intelligence assessments, interviews, and selection centres (Patterson et al. 2016). As of 2020, it is the most widely used test of its kind in the UK, being a compulsory entry requirement for both the UK and international applicants to 31 UK universities, one Non-UK Associate Member University (American University of the Caribbean), and 16 universities across Australia and New Zealand (UKCAT Consortium 2020).

Practice points

- The UCAT cognitive total and verbal reasoning scores have the largest evidence base as weakly positive predictors of academic performance and UK Foundation Programme outcomes.
- Evidence suggests that the UCAT adds a small amount of incremental validity to prior educational attainment.
- Medical schools may wish to deploy cognitive total and verbal reasoning results above other individual subtests in their admissions process.

The UCAT is designed to help universities select applicants ‘with the most appropriate mental abilities, attitudes and professional behaviours required for new doctors and dentists to be successful in their clinical careers’ (UKCAT Consortium 2020, para. 2). The assessment aims to measure performance across a range of mental abilities through its cognitive subtests, including verbal reasoning, decision making, quantitative reasoning, and abstract reasoning. As such, it is intended to serve as a marker of the construct ‘fluid’ intelligence (biologically-based cognitive skills) and potential for achievement, as opposed to that of ‘crystallised’ intelligence (knowledge-based intelligence) through schooling, or prior achievement (e.g. A-levels).

Aptitude testing in this context can be considered as a form of ‘quality assurance,’ as described by Gibbs (1999), in that it provides evidence for those selecting students of their potential for achievement. This notion is further supported by the work of Bloxham and Boyd (2007), who described the role of assessment of learning in enabling judgements to be made about students’ achievements (in the context of an aptitude test, the potential for future achievement) for the purposes of selection and quality assurance.

A fifth element of the test, situational judgment, was introduced in 2013. It assesses non-cognitive traits, specifically the construction of the capacity to understand real-world situations and to identify critical factors and appropriate behaviour in dealing with them (UKCAT Consortium 2020). UCAT scores are used at the discretion of individual universities as part of their selection process. UCAT scores are typically used in one or more of four ways: threshold (most common use—as a threshold to enable an applicant to progress to interview), factor (as one factor among a range of others), borderline (to separate borderline students), and rescue (in a compensatory manner to rescue an otherwise weaker applicant) (Adam et al. 2011).

Selection tools may be judged according to their validity, reliability, acceptability, and practicality (Van Der Vleuten 1996). Criterion validity assesses the correlation between a test and a criterion variable (representative of the construct being assessed and already held to be valid). Criterion validity may be either concurrent or predictive. This review will be concerned specifically with predictive validity; the extent to which a predictor measure (overall UCAT scores and scores on individual subtests) is correlated with a criterion measure obtained at some point in the future. Criterion measures include academic data (such as examination performance) and non-academic outcomes, such as professional behaviour.

Several studies have sought to evaluate the predictive validity of the UCAT as a whole (Husbands et al. 2014), and for specific subtests (Srikathirkamanathan et al. 2017). These studies include both single-institution (Adam et al. 2012) and multi-institution analyses (Curtis and Smith 2020). The criterion measures used also vary, including a range of outcomes from first-year exam results (Wright and Bradley 2010) to fitness to practise declarations (Paton et al. 2018). A systematic review performed in 2018 summarising literature exploring the relationship between UCAT scores and performance in medical and dental school found that the strongest relationships with assessment outcomes were observed for UCAT total score and verbal reasoning, although all relationships were weak (Greatrix et al. 2021). Relationships with skills assessments were weaker than for other assessments (e.g. knowledge or mixed assessments). They noted some evidence suggesting an upwards trend in these relationships over programme years at medical school, with the larger trends observed for UCAT total score, verbal reasoning, and abstract reasoning (Sartania et al. 2014; Tiffin et al. 2016). They identified studies that reported ‘small but significant’ incremental validity of the UCAT over other measures of academic attainment (e.g. A-levels) (McManus et al. 2013; Tiffin et al. 2016).

Other studies have looked at the predictive validity of similar assessments used internationally, such as the Medical College Admissions Test (MCAT) in Canada and the United States of America, and the Graduate Medical School Admissions Test (GAMSAT) in Australia. Studies have shown the ability of the MCAT to predict academic performance in medical school (Dunleavy et al. 2013) and successful performance in post-graduate Canadian and American examinations (Donnon et al. 2007; Raman et al. 2019). Total GAMSAT scores have also been shown to be independent predictors of strong academic performance throughout graduate-entry medical programmes (Puddey and Mercer 2014).

### Aims

The aim of this study is to systematically review the literature for quantitative, qualitative, and mixed-method evidence of the predictive validity of the UCAT. The review aims to answer the following research questions:

1. What is the predictive value of the UCAT across all relevant criterion measures (including academic and non-academic measures of performance both at university and beyond)?
2. What is the comparative predictive validity of total scores and individual subtest scores?
3. Where included in the existing studies, what is the incremental validity of the UCAT when used as an adjunct to other selection tools?

This method of evidence synthesis was chosen to identify, evaluate and summarise relevant studies, thereby making available evidence more accessible to decision-makers. We aim to make recommendations about which UCAT scores might be most fruitful for medical and dental schools to use within their admission procedures and identify areas for further research.

## Materials and methods

### Protocol and registration

The protocol for this review was submitted for registration to the PROSPERO database in October 2019, but was rejected on 6 November 2019 because it was considered out of scope; no further explanation was provided. We believe that since this systematic review focuses primarily on medical education, it was probably not deemed specifically to address a health-related outcome (National Institute for Health Research 2021).

### Inclusion criteria

The UCAT was introduced (as the UKCAT) in 2006. Therefore, studies written in English and published from 2006 onwards were included.A repeat of the search strategy ending in 2006 did not identify any additional studies. Articles were only included where study participants had taken the UCAT. Participants included all prospective medical and dental students, students admitted onto a medical or dental course, and clinicians who had completed medical or dental degrees. It was not necessary for the UCAT to have been deployed as an admissions tool for the study population. Indeed, studies where the UCAT had not been used as a selection criterion, would have the benefit of no range restriction.

Studies were to be considered eligible if they compared the UCAT to one or more of the following predictor variables: other aptitude tests, academic records, personal statements, references, situational judgement tests, personality and emotional intelligence assessments, interviews, and multiple mini‐interviews (MMIs) and selection centres. Studies only considering the UCAT were also included (no comparator).

Only studies assessing criterion validity, specifically predictive validity, were included. Criterion (outcome) measures broadly fell into the following categories: academic results (university level), academic results (post-graduate examinations), performance in situational judgment tests, and other non-academic professional/behavioural outcomes.

Both published and unpublished studies were included in so far as was practicable, including, but not limited to: studies published in peer-reviewed journals, published grey literature studies, studies published on the internet (e.g. on UCAT website), doctoral or undergraduate theses and completed studies awaiting publication.

### Exclusion criteria

Studies assessing construct validity, content validity, test reliability, acceptability, practicality, cost-effectiveness, or stakeholder satisfaction in the absence of predictive validity were omitted. Incomplete studies, conference presentations, or studies in progress were omitted from the systematic review.

### Search strategy

A literature search was conducted on 20 April 2020 using eight electronic databases/sources—MEDLINE, APA PsycInfo, SCOPUS, Web of Science, and grey literature searches using EThOS (British Library), OpenGrey, the official UCAT website, and the PROSPERO website (for any prospectively registered systematic reviews).

Index terms varied across databases but included: UCAT; UKCAT; valid*; predict*; aptitude test*; medical; dental; situational judgement. Medical subject headings (MeSH) were also used where appropriate, including the predictive value of tests, academic performance, professional competence, and school admission criteria. No additional date range limits were applied at the search stage. For example, the electronic search strategy used for the database SCOPUS is below. Strategies and access links for all databases searched can be found in Supplementary Appendix Table 1.

((TITLE- ABS-KEY (ukcat OR ucat OR ¨aptitude test*¨) AND TITLE-ABS-KEY (valid* OR predict*)) AND (LIMIT-TO (AFFILCOUNTRY, ¨United Kingdom¨))

### Screening and study selection

After removal of duplicates, title and abstract screening was undertaken independently by two reviewers (SP or LB, and CB) based on pre-determined eligibility criteria, with disagreements resolved by consensus. Full-text screening (Supplementary Appendix Table 2 for full-text screening tool) was carried out by one of two reviewers (SP or LB) with any studies deemed ineligible cross-checked by a third reviewer (CB).

### Quality appraisal

All 24 eligible studies were cohort studies and quality appraisal was undertaken using the Newcastle-Ottawa Scale for cohort studies by LB (Wells et al. 2011). This quality appraisal tool was chosen since it is a validated scale for assessing quality and risk of bias in observational studies, and has a version specifically for cohort studies (Luchini et al. 2021). All queries were resolved by discussion with CB. In addition, CB checked the quality appraisal outcomes for the four papers with queries and the paper with a ‘poor’ quality rating. The Newcastle-Ottawa scale involves a ‘star system’ where a study is judged in three domains: the selection of the study groups; the comparability of the groups; and ascertainment of the outcome of interest. Within the first quality appraisal domain (selection), four items were assessed: representativeness of the exposed cohort (item 1), selection of the non-exposed cohort (item 2), ascertainment of exposure (item 3), and demonstration that the outcome(s) of interest was not present at the start of the study (item 4). The second domain assessed comparability of cohorts based on adequate study controls (item 5—up to 2 stars). Correcting for restriction in the range where appropriate (i.e. where UCAT scores were used as a selective measure for a cohort) as the most important factor to control for was awarded one star, and additional control variables including gender, age, ethnicity, previous educational attainment were awarded the second star. The third domain of quality appraisal (outcome) analysed how the outcome was assessed (item 6), whether the follow-up was long enough for the outcomes to occur (item 7), and the adequacy of follow-up (item 8).

In accordance with accepted standards, studies were deemed to be of good quality if they scored three or four stars in the selection domain, one or two stars in the comparability domain, and two or three stars in the outcome domain (Agency for Healthcare Research and Quality 2012). Studies were deemed to be of fair quality if they scored two stars in the selection domain, one or two stars in the comparability domain, and two or three stars in the outcome domain. Studies were deemed to be of poor quality if they scored no or one star in the selection domain, or no stars in the comparability domain, or no or one star in the exposure domain.

### Data extraction and synthesis

Data items were extracted from included studies using specifically created data forms and tabulated as results matrices. Data extraction and synthesis were conducted by SP, with a random sample (20%), selected using a random number generator in Excel, checked by a second independent reviewer (LB).

The majority of analyses in the included studies were univariate correlations between the UCAT (intervention) and primary criterion variables (outcomes). A univariate correlations results matrix was created to present all of these analyses, grouped according to primary criterion variable. Findings from analyses of predictor and criterion variables were assigned to one of 24 cell types according to the direction of correlation, effect size, and statistical significance (Table 1). The effect size was determined using Cohen’s rules of thumb (Cohen 1988). With regards to odds ratios, boundaries of OR ≥ 1.50 and OR ≤ −0.66 were set for ‘positive association’ and ‘negative association,’ respectively. Studies with odds ratios between these values were assigned ‘no effect.’ The level of statistical significance was taken as *p* < 0.05.

**Table 1.** Key to correlation results matrices.

Cell type	Description	Effect size
+++ SS	Positive correlation, large effect size, statistically significant	*r* ≥ 0.5
++ SS	Positive correlation, medium effect size, statistically significant	*r* ≥ 0.3
+ SS	Positive correlation small effect size, statistically significant	*r* ≥ 0.1
−−− SS	Negative correlation, large effect size, statistically significant	*r* ≤ −0.5
−− SS	Negative correlation, medium effect size, statistically significant	*r* ≤ −0.3
− SS	Negative correlation, small effect size, statistically significant	*r* ≤ −0.1
+++ NSS	Positive correlation, large effect size, not statistically significant	*r* ≥ 0.5
++ NSS	Positive correlation, medium effect size, not statistically significant	*r* ≥ 0.3
+ NSS	Positive correlation, small effect size, not statistically significant	*r* ≥ 0.1
−−− NSS	Negative correlation, large effect size, not statistically significant	*r* ≤ −0.5
−− NSS	Negative correlation, medium effect size, not statistically significant	*r* ≤ −0.3
− NSS	Negative correlation, small effect size, not statistically significant	*r* ≤ −0.1
Mixed (+ve)	Mixture of any positive SS correlation and not statistically significant and/or no effect	–
Mixed (−ve)	Mixture of any negative SS correlation and not statistically significant and/or no effect	–
No effect SS	No effect, statistically significant	*r* = −0.099–0.099
No effect NSS	No effect, not statistically significant	*r* = −0.099–0.099
Mixed (mixed)	Mixture of any positive and negative correlation (either statistically significant or not statistically significant)	–
+ (OR) SS	Positive association (odds ratio), statistically significant	OR ≥ 1.50
− (OR) SS	Negative association (odds ratio), statistically significant	OR ≤ 0.66
No effect (OR) SS	No effect (odds ratio), statistically significant	OR 0.67–1.49
+ (OR) NSS	Positive association (odds ratio), not statistically significant	OR ≥ 1.50
− (OR) NSS	Negative association (odds ratio), not statistically significant	OR ≤ 0.66
No effect (OR) NSS	No effect (odds ratio), not statistically significant	OR 0.67–1.49
+? SS	Positive correlation but qualitatively (not quantitatively) expressed	–

SS: statistically significant; NSS: not statistically significant; OR: Odds ratio; *r*: correlation coefficient.

If an aggregate result was available which precisely matched any of the primary criterion variables, this was preferred over individual criterion measures (such as results from several specific examinations). Where no aggregate result was available, the individual criterion measures within each primary criterion variable were analysed. Where individual criterion measures were unanimously positive/negative/no effect AND unanimously statistically significant/not statistically significant, an unweighted mean was taken of the correlation coefficients and the appropriate positive correlation/negative correlation cell type was assigned based on the unweighted mean. If results of individual criterion measures were not unanimous, a mixed cell type was assigned as described in Table 1.

Results for the primary variables ‘pre-clinical examination total’ and ‘clinical examination total’ were not separated from their corresponding knowledge-based and skills-based primary criterion variables, since it was deemed more informative to present the knowledge-based and skills-based results separately. Only where studies used pre-clinical or clinical totals as a criterion measure (or where the data forced this grouping) were they added to the results matrix. Hence, several studies have a knowledge-based examination and skills-based examination results, but not pre-clinical total/clinical total. Course academic total was, similarly, not extrapolated from pre-clinical total and clinical total, and UK Foundation Programme (UKFPO) score total not extrapolated from UKFPO Educational Performance Measure (EPM) and UKFPO SJT.

Where studies had results both corrected and uncorrected for range restriction, corrected data was used. Studies were grouped by predictor variable and arranged in descending order of study population size. Criterion variables were presented in chronological order from left to right. Course completion and fitness to practice declarations were included in the matrix (despite being binary variables and therefore not expressed as correlation coefficients) since they were defined as primary criterion variables at the outset.

The primary criterion variable ‘professional behaviour in medical school (clinical)’ is described as ‘adverse outcomes’ in the results matrix because the outcome was evaluated as a negative (poor professional behaviour). Similarly, ‘FtP declarations at registration—health’ and ‘FtP declarations at registration—conduct’ are deemed negative outcomes, and therefore a ‘good’ odds ratio would be OR ≤ 0.66, coded ‘− OR.’ A ‘good’ correlation between a predictor and this criterion would therefore be a negative one.

Where studies only presented multivariate analysis results, these were tabulated separately. A narrative synthesis was then performed, making general observations before interpreting the data grouped by criterion variable. A narrative description of studies evaluating incremental validity was also undertaken.

## Results

### Study selection

**Figure 1.**
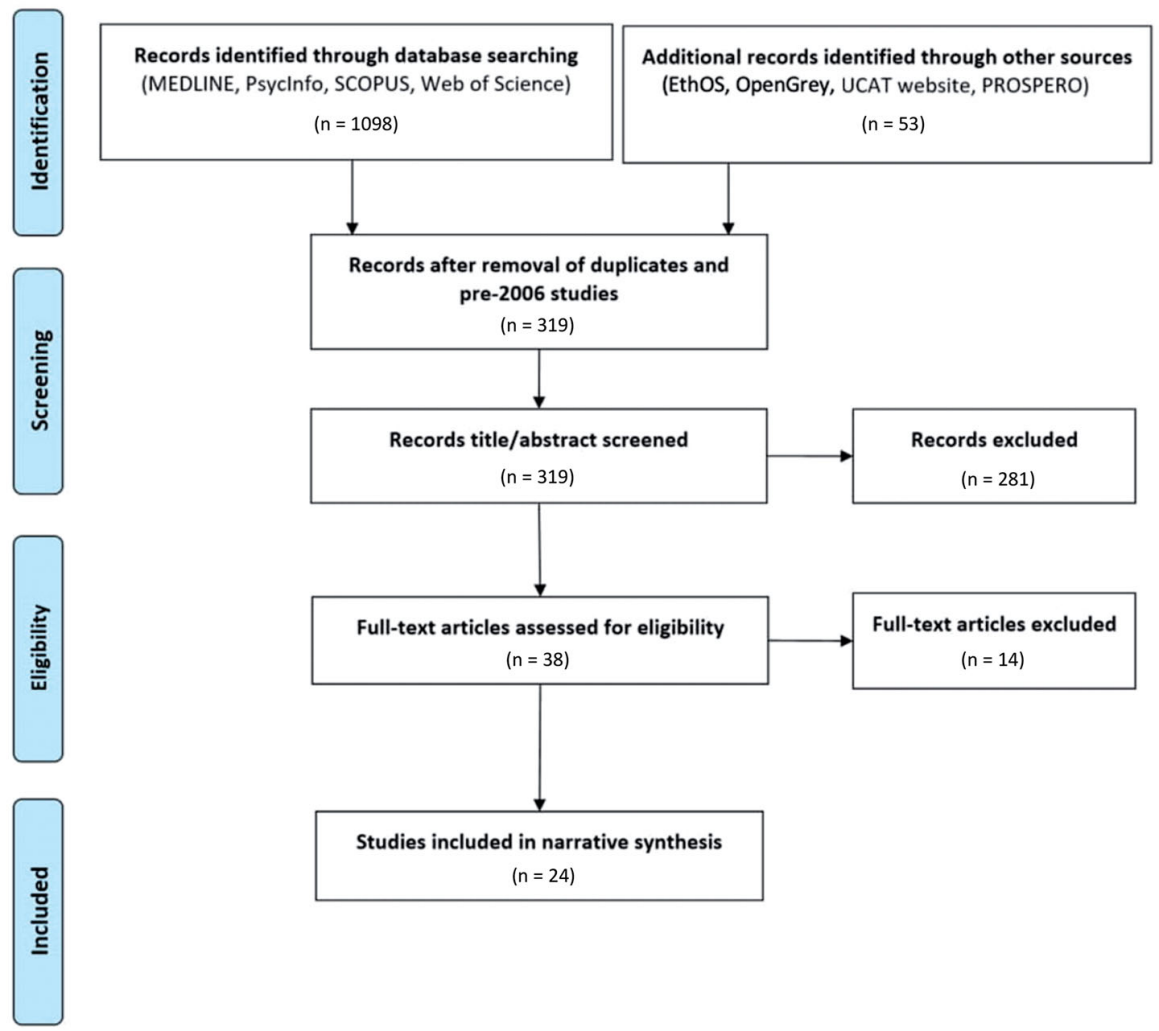
PRISMA flow diagram of search and study selection process.

Our literature search yielded a total of 1151 articles. Three hundred and nineteen articles remained after the removal of duplicates and pre-2006 studies (before the introduction of the UCAT). Two hundred and eighty-one articles were excluded following title and abstract screening (inter-rater reliability *κ* = 0.637), with the remaining 38 studies proceeding to full-text screening. Reasons for the exclusion of studies at the full-text screening stage are documented in Supplementary Appendix Table 3. Twenty-four studies were subsequently identified as meeting the inclusion criteria, as depicted in the PRISMA flow diagram (Figure 1), and were all cohort studies.

### Study characteristics

The characteristics of the 24 included studies are summarised in Supplementary Appendix Table 4. Study type, sample size, institutions, course details, years of UCAT data, predictor variables, criterion measures, and whether correction for restriction in range and/or multiple imputations for missing data was used.

### Quality appraisal

The results of the quality appraisal of the 24 included studies are detailed in Supplementary Appendix Table 5. Twenty-three of the 24 studies were assessed to be of good quality. Only one study (Lynch et al. 2009) was deemed to be of poor quality. Lynch’s study aimed to identify whether the UCAT total and subset scores could predict Year 1 outcomes for 297 medical undergraduates at the University of Aberdeen and the University of Dundee. This study was assessed to be of poor quality due to the absence of a correction for restriction in range and the absence of relevant control variables in the multiple regression analysis. Although UCAT scores were not used for admissions by the University of Aberdeen for the 2007–2008 cohort studied, the University of Dundee used the UCAT total score to rank applicants (applicable to 60 out of 167 applicants at Dundee) near the cut‐point for offers, and therefore a correction for restriction in range should have been applied to the data. Furthermore, this study did not control for any demographic variables (e.g. gender, age, and ethnicity) or previous educational attainment, although the authors state that the demographic profiles of the students suggested no obvious departure from the national profile.

### Correlations results matrix

There was a considerable degree of heterogeneity between included studies in terms of the specific criterion measures assessed. For example, each institution had varying examination structures and/or measures of professional behaviour. Across the range of selected studies, examination results were differentially delineated according to year, theme, type (written/practical), and knowledge-based/skills-based, or simply divided into each individual examination. Moreover, some studies had corrected for restriction in range and used multiple imputations of data whereas others had not. We addressed this heterogeneity by categorising predictor variables into UCAT subtests and total scores, and outcomes variables into pre-clinical *vs.* clinical assessments, knowledge *vs.* skills-based assessments, course completion, measures of professional behaviour, UKFPO outcomes, and fitness to practice declarations. Supplementary Appendix Table 6 (univariate) and Supplementary Appendix Table 7 (multivariate) show the individual study results classified into the 24 cell types as shown in Table 1.

### Results matrix: Distribution of data

The univariate results matrix contained a total of 192 data points, the distribution of which according to predictor and criterion variables is summarised in Table 2. The multivariate results matrix had four data points (Supplementary Appendix Table 7). The cognitive total had the most data available, followed by the individual cognitive subtests. There were scant data available for the predictive validity of the SJT subtest and cognitive + SJT total, with only six studies including the SJT in their analyses. This is likely explained by the SJT not having been introduced as a UCAT subtest until 2013. Overall, the quantity of data was found to decrease with increasing length of follow-up. Pre-clinical examinations had the most data points, followed by clinical examinations, with a sharp decline in the number of data points for professional behaviour/UKFPO measures and even fewer for fitness to practice declarations at registration.

**Table 2.** Distribution of univariate data points according to predictor and criterion variables.

Predictor variables	Cognitive total	Cognitive + SJT total	AR	DA	QR	VR	SJT	
	*n* = 49	*n* = 4	*n* = 35	*n* = 31	*n* = 32	*n* = 35	*n* = 6	
Criterion variables	Pre-clinical knowledge-based	Pre-clinical skills-based	Pre-clinical total	Clinical knowledge-based	Clinical skills-based	Clinical total	Professional behaviour in MS (pre-clinical)	Professional behaviour in MS (clinical—adverse outcomes)
	*n* = 41	*n* = 29	*n* = 15	*n* = 24	*n* = 25	*n* = 5	*n* = 9	*n* = 4
	Course academic total	Course completion	UKFPO EPM total	UKFPO EPM decile	UKFPO SJT	UKFPO total	FtP declarations (health)	FtP declarations (conduct)
	*n* = 7	*n* = 1	*n* = 10	*n* = 7	*n* = 7	*n* = 5	*n* = 1	*n* = 2

### Results matrix: Interventions

Fifty-three out of 192 univariate data points and two out of four multivariate data points (total 55/196 or 28%) were ‘good’ statistically significant correlations with at least a small effect size (positive for desirable outcomes, negative for adverse outcomes). Most were of weak effect size (± SS, *n* = 50 univariate data, *n* = 2 multivariate data), few were of medium effect size (++/−− SS, *n* = 3), and none were of large effect size (+++/−−− SS, *n* = 0). This may partly be explained by the fact that some studies had not corrected for restriction in range despite the UCAT having been used as an admissions tool in the selection of the study population. This practice would tend to diminish the apparent predictive validity.

Based on the number of data points alone, predictors were ranked in terms of which showed the greatest proportion of positive SS correlations or mixed (+ve) correlations, or the inverse for adverse outcomes. The cognitive total and verbal reasoning subset showed the most evidence for predictive validity across all outcome measures, with abstract reasoning showing the least (Table 3).

**Table 3.** Ranking of predictor variables by ‘good’ correlations for univariate data.

Rank	Predictor variable	*N* (+ve SS or mixed + ve)/*n* (data points for given predictor variable) (%)
1	Cognitive total	35/49 (71%)
2	VR	19/35 (54%)
3	SJT	3/6 (50%)
4	DA	13/31 (42%)
5	QR	10/32 (31%)
6	Cognitive + SJT total	1/4 (25%)
7	AR	7/35 (20%)

### Results matrix: Interpretation by outcome

#### Pre-clinical examinations

The cognitive total and VR subset were most effective at predicting pre-clinical examinations, particularly pre-clinical knowledge examinations. These findings were often based on studies that had very large numbers of participants (e.g. 14,379 participants in Paton et al. 2018 study). Two of the three medium effect size correlations in this review involved pre-clinical exams for the predictor cognitive total (the other for the SJT). AR fared poorly; only Adam et al. (2012) (not subject to range restriction) showed a weak correlation between abstract reasoning and pre-clinical total, the remainder showing no effect. Tiffin et al. (2016), a large study (*n* = 6812), showed that the cognitive total and all cognitive subtests predicted pre-clinical knowledge examinations, with the exception of AR. DA and QR showed a mixture of weak correlations and no effect on pre-clinical examination performance.

#### Clinical examinations

Cognitive total and VR showed the greatest predictive validity. 12/15 data points for cognitive total and 8/10 for VR were either + SS or mixed (+ve). AR again showed poor predictive validity, with only Adam et al. (2015) (not subject to range restriction) showing a weak positive correlation with clinical knowledge-based and skills-based exams, the remainder of data points showing no effect. DA and QR showed a mixture of weak correlations and no effect for clinical examinations.

#### Professional behaviour in medical school

The cognitive subtests predicted both desirable and adverse outcomes in some, but not all pre-clinical professionalism assessments, amounting to a mixed picture. However, as would be expected, the SJT showed slightly better predictive ability in the two studies by Patterson et al., which were medium-sized studies surveying two to three medical schools. A single study (Adam et al. 2015) showed mixed (−ve) correlations with adverse outcomes in the clinical phase for cognitive total, AR and QR (negative being a ‘good’ correlation in this case). This indicates that those subtests predicted positive outcomes in some, but not all assessments of professional behaviour.

#### Course academic total

Only cognitive + SJT total percentile was found to have a weak predictive validity for whole-course academic performance.

#### Course completion

A single paper (Garrud and McManus 2018) described in qualitative terms an association between cognitive total and course completion.

#### UKFPO

Data points across the spectrum of UKFPO criterion variables were weakly positive statistically significant correlations or qualitatively positive correlations in 20/29 cognitive test data points. In MacKenzie et al. (2016), cognitive total and VR were unanimously weakly predictive across all four primary UKFPO criterion variables.

#### Fitness to practice declarations

There was no evidence of the predictive ability of the UCAT to predict either health- or conduct-related fitness to practice declarations at GMC registration.

### Outcomes for dental students

Data for dental schools was limited, with only five out of 24 included studies reporting findings for dental students. Lala et al. (2013) only found significant correlations between the decision analysis subtest score and first-year examination performance in dental school (first-semester performance *r* = 0.203, *p* < 0.05, second-semester performance *r* = 0.179, *p* < 0.05). Foley and Hijazi (2015) demonstrated a significant relationship between total UCAT score and UCAT percentile with a combined university assessment score taking into account examinations across 4 years of graduate dental school (*r*^2^ = 0.077, *p* = 0.019 and *r*^2^ = 0.118, *p* = 0.001, respectively). Lambe et al. (2018) reported a statistically significant relationship between total UCAT scores in first-year assessments (*r* = 0.32, *p* < 0.01, 3 = 0.38. *p* < 0.05), but not at the individual UCAT subtest level. McAndrew et al. (2017) found no correlation between UCAT scores and first-year examination performance. Further analysis of performance by grade boundaries (1st, 2i, 2ii 3rd, fail, etc.) identified a significant association between total UCAT score and poor examination performance for those obtaining a 3rd, borderline fail, or fail (*p* = 0.06 and *p* = 0.03 for Cardiff, *p* = 0.001 for Newcastle). Patterson et al. (2017) reported significant correlations between SJT scores and both mean supervisor ratings (uncorrected *r* = 0.24, *p* < 0.001; corrected *r* = 0.34) and overall judgments (uncorrected *r*s = 0.16, *p* < 0.05; corrected *r*s = 0.20) for professional behaviours (integrity, perspective taking and team involvement). However, this study did not differentiate between medical (*n* = 197) and dental (*n* = 21) students and dental-specific outcomes were not reported.

### Incremental validity

Two studies considered the incremental validity of the UCAT after controlling for prior educational attainment (McManus et al. 2013; Tiffin et al. 2016). McManus et al. demonstrated a statistically significant improvement, but a small effect (beta coefficient 0.057) for the effect of the cognitive total score on pre-clinical total scores, whilst controlling for prior educational attainment. Tiffin et al. demonstrated that many of the associations with criterion measures remained statistically significant despite controlling for the influence of prior educational attainment. Thus, the UCAT can be assumed to add incremental value above and beyond that provided by prior educational attainment (e.g. actual or predicted A-level or equivalent grades). Of note, the incremental value was not quantified in Tiffin et al.’s study, but based on assumption.

## Discussion

This study provides an up-to-date synthesis of outcomes from articles reporting on the predictive validity of the UCAT. Individual universities use UCAT scores in different ways as part of their selection process, often citing varying minimum overall or subtest scores (e.g. the SJT) to proceed to interview, or as one factor amongst a range of others to select students for interview. Universities also differ in the weighting attributed when factoring the UCAT into their selection process.

Findings from this review demonstrated that for over 70% of data points, the UCAT exerted no statistically significant predictive validity in the direction sought. This may to some extent be attributable to range restriction in the included studies. Where the UCAT does exhibit predictive validity, its predictive power is weak, or its predictive effect is blunted because it predicts success in only certain relevant outcomes. This data highlights the limitations of point-in-time high-stakes assessments for the purposes of application selection for medical school.

Given that cognitive total and verbal reasoning subtest scores showed the most evidence of predictive validity across all outcome measures (including UKFPO outcomes), medical schools could consider using cognitive total and verbal reasoning scores over and above other individual subtests, with the objective of predicting in-course academic performance and UKFPO success.

There is some evidence that the situational judgment subtest weakly predicts professional behaviour in medical school, whilst the cognitive subtests did not. Only cognitive + SJT total percentile was found to have a weak predictive validity for whole-course academic performance. Where professional behaviour is a primary concern for medical schools, they may wish to consider this individual subtest result.

There is a small amount of evidence for the incremental validity added by the UCAT to prior educational attainment as a selection measure. The effect appears to be small and further research is required to ascertain the UCAT’s incremental validity with other admissions tools. There was a paucity of studies looking at post-graduate outcomes, with only 1 study demonstrating that UCAT scores did not predict health- and conduct-related fitness to practice declarations at GMC registration. Similarly, only five out of 24 studies reported outcomes for dental students, one of which was a mixed study that did not differentiate between medical and dental students (Patterson et al. 2017). Significant findings were inconsistent across the 5 studies, with some demonstrating a significant relationship between total UCAT score and first-year assessments (Lambe et al. 2018) and others findings no significant relationship (McAndrew et al. 2017). One study found a significant relationship between the DA subtest and first-year examination performance (Lala et al. 2013), whereas the others did not.

The limitations of this systematic review include that the DA subtest no longer features in the UCAT. In 2016, a new Decision-Making subtest was piloted and from 2017, was included in UCAT scores. No studies had, at the time of writing, assessed the predictive validity of this new subtest, and the absence of DA may limit the applicability of our cognitive total analyses to the present UCAT test. Of the included studies in this review, many are small and only a minority collect data from three or more cohorts or institutions. This will inevitably impact upon the strength of the evidence, although the vast majority were of good quality. The univariate results matrix in this study used unweighted means and did not take into account the weighting of individual exams or assessment components. Furthermore, the quantity of data available decreases sharply with increasing length of follow-up; the evidence is therefore heavily weighted towards the early years of study.

Further research into the predictive validity of the UCAT is needed to address these limitations, such as analysing data from 2017 candidates onwards to assess the predictive validity of the Decision-Making subtest. The UCAT is primarily a university admissions tool and further exploration is needed to ascertain the incremental validity of the UCAT over other admission tools. Further research is also needed for dental students and to investigate post-graduate outcomes beyond professional registration, not least since the recruitment of students who are going to make good doctors and dentists, as opposed to high-performing students, is arguably a worthwhile aim in a highly vocational course. Such studies will become increasingly feasible as cohorts who have sat the UCAT progress through the medical and dental professions.

## Conclusions

The UCAT cognitive total and verbal reasoning subtest have the largest evidence base as weak positive predictors of clinical and pre-clinical academic performance and UKFPO outcomes. Hence, medical schools could deploy these over and above other individual subtests when selecting medical students, with the objective of predicting in-course academic performance and UKFPO success. There is some evidence that the situational judgment subtest weakly predicts professional behaviour in medical school; where this is a primary concern for medical schools they may wish to consider this individual subtest result. There is a small amount of evidence for the incremental validity added by the UCAT to prior educational attainment as a selection measure. The effect appears to be small and further research is required to ascertain the UCAT’s incremental validity with other admissions tools.

## Supplementary Material

Supplemental Material

## Glossary

**University Clinical Aptitude Test (UCAT):** An admissions assessment used by a consortium of universities across the UK, Australia, and New Zealand, to aid the selection of applicants to medical and dental degree programmes. The UCAT aims to measure mental aptitude and professional behaviours required to become successful doctors and dentists (UKCAT Consortium 2020).
