# Supplementary material for: Assessing the predictive validity of the UCAT—A systematic review and narrative synthesis
Source: Med Teach. 2021 Nov 23;44(4):401–9. doi: 10.1080/0142159X.2021.1998401 (PMC9162495; doi:10.1080/0142159X.2021.1998401)
Supplement: Supplemental Material [file IMTE_A_1998401_SM6524.docx]

**Appendix**

| **Database** | **Access Link** | **Search Strategy** |
| --- | --- | --- |
| MEDLINE | https://www.wolterskluwer.com/en/solutions/ovid/ovid-medline-901 | (ucat OR ukcat) OR ((Aptitude Tests OR ukcat OR ucat or situational judgement) AND (exam performance OR predict* OR valid* OR academic performance OR professional competence OR school admission criteria)) |
| APA PsycInfo | https://www.apa.org/pubs/databases/psycinfo | (ucat OR ukcat) OR ((exp Student Admission Criteria/ or exp Aptitude Measures OR ukcat OR ucat) AND (exp Statistical Validity/ or exp Criterion Validity/ or exp Predictive Validity/ or exp Test Validity)) |
| SCOPUS | https://www.elsevier.com/en-gb/solutions/scopus | (( TITLE-ABS-KEY ( ukcat  OR  ucat  OR  "aptitude test*" ) AND  TITLE-ABS-KEY ( valid*  OR  predict* ) )  AND  ( LIMIT-TO ( AFFILCOUNTRY ,  "United Kingdom" ) ) |
| Web of Science | https://clarivate.libguides.com/webofscienceplatform/alldb | TS=(UKCAT OR UCAT OR ("aptitude test*" AND (MEDICAL OR DENTAL))) AND TS=( valid* OR predict*) |
| EThOS | https://ethos.bl.uk/Home.do | ucat or ukcat |
| OpenGrey | http://www.opengrey.eu | ucat or ukcat |
| UCAT website | https://www.ucat.ac.uk/about-us/published-research/ | ucat or ukcat |
| PROSPERO website | https://www.crd.york.ac.uk/prospero/ | ucat or ukcat |

Appendix Table 1: Electronic search strategies for databases

| **Criterion** | **Yes** | **No (Exclude)** |
| --- | --- | --- |
| Completed study available/published |  |  |
| Publication date after or including 2006 |  |  |
| Study in English language |  |  |
| Population: prospective medical and/or dental students |  |  |
| Intervention: UCAT score used as a predictor variable |  |  |
| Control: Score on any other predictor variable from an assessment in the same academic year as the UCAT/none |  |  |
| Outcome: Any measure of performance at medical or dental school or after graduation in clinical practice (i.e. predictive validity) |  |  |
| Study design: Cohort – must follow-up from taking UCAT to performance in medical or dental school or beyond (even if not labelled as such) |  |  |

Appendix Table 2: Full-text screening tool

| **Author, year** | **Title** | **Reason for exclusion** | **Details** |
| --- | --- | --- | --- |
| Cunningham et al., 2019 | A literature review of the predictive validity of European dental school selection methods | Duplication | Individual studies in paper already included in this systematic review |
| Finn et al., 2018 | The ability of 'non-cognitive' traits to predict undergraduate performance in medical schools: a national linkage study | Intervention | Used piloted tests not incorporated into UCAT |
| Harrington et al., 2018 | Selection criteria to surgical residency: predictors of performance. A systematic review and meta-analysis | Publication status | Systematic review protocol – review still in progress |
| Kumwenda et al., 2013 | Is embellishing UCAS personal statements accepted practice in applications to medicine and dentistry | Outcomes | Not assessing the predictive validity of the UCAT |
| Kumwenda et al., 2017 | The relationship between school type and academic performance at medical school: a national, multi-cohort study | Intervention, outcomes | Uses UCAT as a criterion measure, not as a predictor variable |
| MacKenzie et al., 2017 | Do personality traits assessed on medical school admission predict exit performance? A UK-wide longitudinal cohort study | Intervention | Piloted tests not incorporated into UCAT |
| McManus et al., 2013 | The UKCAT-12 Study Technical Report Educational attainment, aptitude test performance, demographic and socio-economic contextual factors as predictors of first year outcome in twelve UK medical schools | Duplication | Technical report, main study included |
| McManus et al., 2013 | Construct-level predictive validity of educational attainment and intellectual aptitude tests in medical student selection: meta-regression of six UK longitudinal studies | Outcomes | Deals with construct validity, not criterion validity |
| McManus et al., 2013 | The Academic Backbone… longitudinal continuities in educational achievement from secondary school and medical school to MRCP(UK) and the specialist register in UK medical students and doctors | Intervention | UCAT not used as a predictor variable |
| Patterson et al., 2016 | How effective are selection methods in medical education? A systematic review | Duplication | Qualitative synthesis only (No primary data, no meta-analysis). Four included UCAT-related studies used are included already included in this systematic review |
| Tiffin et al., 2018 | Artificial or intelligent?  Machine learning and medical selection: possibilities and risks | Outcomes, duplication | Predictive validity not discussed. Dataset duplicated in Mwandigha et al., 2018 (already included in this systematic review) |
| Tiffin et al., 2019 | Does 'online confidence' predict application success and later academic performance in medical school? A UK-based national cohort study. | Intervention | Confidence with UCAT scores, not actual UCAT scores used as a predictor variable |
| Webster et al., 2019 | Situational judgement tests for medical selection: a systematic review and meta-analysis | Publication status | Systematic review protocol – review still in progress |
| Wright, 2012 | A mixed methods study of medical school admissions : issues of fairness and student performance | Duplication | PhD thesis, full text obtained from author. Author confirmed thesis duplicates Wright et al., 2010 (already included in this systematic review) |

Appendix Table 3: Reasons for the exclusion of studies at the full-text screening stage

| **Author, date** | **Study Title** | **Study Type** | **Sample size** | **Course** | **Data Source** | **UCAT year** | **Predictor variables** | **Criterion measures** | **Multiple imputation for missing data** |
| --- | --- | --- | --- | --- | --- | --- | --- | --- | --- |
| Adam et al., 2012 | Can personal qualities of medical students predict in-course examination success and professional behaviour? An exploratory prospective cohort study | Cohort | 122 | Undergraduate medicine | Hull York Medical School | 2006 | AR, DA, QR, VR, cognitive total | Pre-clinical total, professional behaviour in medical school | No |
| Adam et al., 2015 | Predictors of professional behaviour and academic outcomes in a UK medical school: A longitudinal cohort study | Cohort | 131 | Undergraduate medicine | Hull York Medical School | 2006 | AR, DA, QR, VR, cognitive total | Clinical knowledge-based, clinical skills-based, professional behaviour in medical school | No |
| Curtis et al., 2020 | A comparison of undergraduate outcomes for students form gateway courses and standard entry medicine courses | Cohort | 4205 | Undergraduate medicine | King’s College London, Southampton and Norwich | Kings: 2008 – 2012  Southampton 2008 - 2012 | Cognitive total | UKFPO EPM, UKFPO SJT, PSA, clinical knowledge-based exam (2014 onwards) | No |
| Foley et al., 2015 | Predictive value of the admissions process and the UK Clinical Aptitude Test in a graduate-entry dental school | Cohort | 71 | Graduate-entry dentistry | Aberdeen | 2009-2013 | Cognitive and SJT total, UCAT total percentile (relative to others who sat UKCAT in same year) | Course academic total | No |
| Garrud et al., 2018 | Impact of accelerated, graduate-entry medicine courses: a comparison of  profile, success, and specialty destination between graduate entrants to  accelerated or standard medicine courses in UK | Cohort | 2030 | Graduate-entry and undergraduate medicine | UKMED database | 2006, 2007 | cognitive total | Course completion, UKFPO EPM, UKFPO SJT | Yes |
| Husbands et al., 2013 | Predictive validity of the Dundee multiple mini-interview | Cohort | 418 | Undergraduate medicine | Dundee | 2009, 2010 | Cognitive total | Pre-clinical knowledge-based, pre-clinical skills-based, clinical-knowledge, clinical skills-based | No |
| Husbands et al., 2014 | Predictive validity of the UK clinical aptitude test in the final years of medical school: a prospective cohort study | Cohort | 239 | Undergraduate medicine | Aberdeen, Dundee | 2006 | Cognitive total | Clinical knowledge-based, clinical skills-based | No |
| Lala et al., 2013 | Validity of the UKCAT in Applicant Selection and Predicting Exam Performance in UK Dental Students | Cohort | 127 | Undergraduate dentistry | Sheffield | 2007, 2008 | AR, DA, QR, VR, cognitive total | Pre-clinical knowledge-based | No |
| Lambe, 2018 | Exploring uses of the UK Clinical Aptitude Test-situational judgement test in a dental student selection process | Cohort | 228 | Undergraduate dentistry | Peninsula | 2013 | AR, DA, QR, VR, cognitive total, SJT | Pre-clinical knowledge-based, pre-clinical skills-based | No |
| Lynch et al., 2009 | Does the UKCAT predict Year 1 performance in medical school? | Cohort | 297 | Undergraduate medicine | Aberdeen, Dundee | 2006 | AR, DA, QR, VR, cognitive total | Pre-clinical knowledge-based, pre-clinical skills-based | No |
| MacKenzie et al., 2016 | Does the UKCAT predict performance on exit from medical school?  A national cohort study | Cohort | 6294 | Graduate-entry and undergraduate medicine | 30 medical schools | 2006, 2007, 2008 | AR, DA, QR, VR, cognitive total | UKFPO EPM (decile), UKFPO EPM (total), UKFPO SJT, UKFPO total | No |
| McAndrew et al., 2016 | Does a selection interview predict year 1 performance in dental school? | Cohort | 177 | Undergraduate dentistry | Cardiff, Newcastle | unclear | Cognitive and SJT total | Pre-clinical knowledge-based, pre-clinical skills-based | No |
| McManus et al., 2013 | The UKCAT-12 study: educational attainment, aptitude test performance, demographic and socio-economic contextual factors as predictors of first  year outcome in a cross-sectional collaborative study of 12 UK medical schools | Cohort | 4811 | Undergraduate medicine | 12 medical schools | 2006, 2007, 2008 | AR, DA, QR, VR, cognitive total | Pre-clinical knowledge-based, pre-clinical skills-based, pre-clinical total | Yes |
| Mwandigha et al., 2018 | What is the effect of secondary (high) schooling on subsequent medical  school performance? A national, UK-based, cohort study | Cohort | 2107 | Undergraduate medicine | 18 medical schools | 2007 | Cognitive total | Pre-clinical knowledge-based, pre-clinical skills-based, clinical knowledge-based, clinical skills-based | Yes |
| Paton et al., 2018 | Predictors of fitness to practise declarations in UK medical undergraduates | Cohort | 14379 | Undergraduate medicine | UKMED database | 2006, 2007 | AR, DA, QR, VR, cognitive total | FtP declarations at registration – health and conduct | No |
| Patterson et al., 2014 | UKCAT SJT: a study to explore validation methodology and early findings | Cohort | 310 | Undergraduate medicine | 2 medical schools | 2012 (Pilot SJT) | SJT | Professional behaviour in medical school (performance questionnaires) | No |
| Patterson et al., 2017 | The Predictive Validity of a Text-Based Situational Judgment Test in Undergraduate Medical and Dental School Admissions | Cohort | 218 | Undergraduate medicine and dentistry | 3 medical schools, 1 dental school | 2013 | SJT | Professional behaviour in medical school | No |
| Sartania et al., 2014 | Predictive power of UKCAT and other pre-admission measures for performance in a medical school in Glasgow: a cohort study | Cohort | 189 | Undergraduate medicine | Glasgow | 2006 | AR, DA, QR, VR, cognitive total | Pre-clinical knowledge-based, clinical knowledge-based, clinical skills-based, course academic total, EPM total | No |
| Srikathirkamanathan et al., 2017 | The relationship between UKCAT scores and Finals exam performance for widening access and traditional entry students | Cohort | 183 | Undergraduate medicine | Southampton | 2007, 2008 | AR, DA, QR, VR, cognitive total | Clinical knowledge-based, clinical skills-based, clinical total | No |
| Tiffin et al., 2016 | Predictive validity of the UKCAT for medical school undergraduate performance: a national prospective cohort study | Cohort | 6812 | Undergraduate medicine | 18 medical schools | 2006, 2007 | AR, DA, QR, VR, cognitive total | Pre-clinical knowledge-based, pre-clinical skills-based, clinical knowledge-based, clinical skills-based | Yes |
| Tiffin et al., 2017 | Exploring the validity of the 2013 UKCAT SJT- prediction of undergraduate performance in the first year of medical school: Summary Version of Report | Cohort | 1383 | Undergraduate medicine | 8 medical schools | 2013 | AR, DA, QR, VR, cognitive total, SJT | Pre-clinical knowledge-based, pre-clinical skills-based | No |
| Wright et al., 2010 | Has the UK Clinical Aptitude Test improved medical student selection? | Cohort | 307 | Undergraduate medicine | Newcastle | 2006, 2007 | Cognitive total | Clinical total | No |
| Yates et al., 2010 | The value of the UK Clinical Aptitude Test in predicting pre-clinical performance: a prospective cohort study at Nottingham Medical School | Cohort | 204 | Undergraduate medicine | Nottingham | 2006 | AR, DA, QR, VR, cognitive total | Pre-clinical knowledge-based, pre-clinical skills-based | No |
| Yates et al., 2013 | The UK clinical aptitude test and clinical course performance at Nottingham: a prospective cohort study | Cohort | 204 | Undergraduate medicine | Nottingham | 2006 | AR, DA, QR, VR, cognitive total | Clinical knowledge-based, clinical skills-based | No |

Appendix Table 4: Summary of Study Characteristics. UKMED: UK Medical Education Database, AR: Abstract Reasoning, DA: Decision Analysis, QR: Quantitative Reasoning, VR: Verbal Reasoning, FtP: Fitness to Practise, UKFPO: UK Foundation Programme, EPM: Educational Performance Measure, SJT: Situational Judgement Test

|  | **Selection** | | | | **Comparability** | **Outcome** | | |  |  |
| --- | --- | --- | --- | --- | --- | --- | --- | --- | --- | --- |
| **Author, year** | **Item 1** | **Item 2** | **Item 3** | **Item 4** | **Item 5 (*RiR)** | **Item 6** | **Item 7** | **Item 8** | **Comments** | **Overall quality** |
| Adam et al., 2012 | * | * | * | * | *(RiR N/A) | * | * | * | Item 5 – UKCAT not used for student selection | Good |
| Adam et al., 2015 | * | * | * | * | **(RiR N/A) | * | * | * | Item 5 – UKCAT not used for student selection | Good |
| Curtis et al., 2020 | * | * | * | * | * | * | * | * | Item 5 – control for previous educational attainment/ A-levels. No RiR correction | Good |
| Foley et al., 2015 | * | * | * | * | * | * | * | * | Item 5 – no RiR correction | Good |
| Garrud et al., 2018 | * | * | * | * | * | * | * |  | Item 5 – no RiR correction  Item 8 – Multiple imputation required since information incomplete at many stages. Only some LtFU explained (e.g. 2007 entrants on 4 year course did not sit SJT). LtFU 14% for EPM and 67% for SJT | Good |
| Husbands et al., 2013 | * | * | * | * | ** | * | * | * |  | Good |
| Husbands et al., 2014 | * | * | * | * | * | * | * | * | Item 5 – no RiR correction | Good |
| Lala et al., 2013 | * | * | * | * | * | * | * | * | Item 5 – no RiR correction | Good |
| Lambe, 2018 | * | * | * | * | * (RiR N/A) | * | * | * | Item 5 – UKCAT not used for student selection | Good |
| Lynch et al., 2009 | * | * | * | * |  | * | * | * | Item 5 – no RiR correction or required control variables | **Poor** |
| MacKenzie et al., 2016 | * | * | * | * | * | * | * | * | Item 5 – no RiR correction | Good |
| McAndrew et al., 2016 | * | * | * | * | * | * | * | * | Item 5 – no RiR correction | Good |
| McManus et al., 2013 | * | * | * | * | * | * | * | * | Item 5 – no RiR correction | Good |
| Mwandigha et al., 2018 | * | * | * | * | * | * | * |  | Item 5 – no RiR correction  Item 8 – over 30% LtFU for 1^st^ year UG results and increasing loss for subsequent years (% attrition up to 75%) | Good |
| Paton et al., 2018 | * | * | * | * | * |  | * | * | Item 5 – no RiR correction  Item 6 – self-reported FtP issues | Good |
| Patterson et al., 2014 | * | * | * | * | *(RiR N/A) | * | * |  | Item 5 – no control variables  Item 8 – outcome data from 310 students, but SJT scores only from 219 students with no explanation (29% LtFU) | Good |
| Patterson et al., 2017 | * | * | * | * | * | * | * | * | Item 5 – RiR correction applied, but no other relevant control variables | Good |
| Sartania et al., 2014 | * | * | * | * | * | * | * | * | Item 5 – no RiR correction | Good |
| Srikathirkamanathan et al., 2017 | * | * | * | * | *(RiR N/A) | * | * | * | Item 5 – UKCAT not used for student selection. No relevant control variables | Good |
| Tiffin et al., 2016 | * | * | * | * | ** | * | * |  | Item 8 – 64% attrition rate for 5^th^ year UG student data from 2008 UKCAT sitting | Good |
| Tiffin et al., 2017 | * | * | * | * | ** | * | * | * |  | Good |
| Wright et al., 2010 | * | * | * | * | * | * |  | * | Item 5 – no RiR correction  Item 7 – Not enough time for follow up Year 2 examination score data only available for cohort 1 (starting med school 2007/8), but not for cohort 2 (starting med school 2008/9) | Good |
| Yates et al., 2010 | * | * | * | * | * | * | * | * | Item 5 – no RiR correction | Good |
| Yates et al., 2013 | * | * | * | * | * | * | * | * | Item 5 – no RiR correction  Item 8 – 9% loss to F/U (204 to 185) | Good |

Appendix Table 5: Quality appraisal of selected studies using the Newcastle-Ottawa scale. RiR: Restriction in range. LtFU: Loss to follow up. UG: undergraduate. FtP: Fitness to practise.

| **UCAT Subtest** | **Study** | **Pre-clinical knowledge-based** | **Pre-clinical skills-based** | **Pre-clinical total** | **Clinical knowledge-based** | **Clinical skills-based** | **Clinical total** | **Prof behaviour in MS (pre-clinical)** | **Professional behaviour in MS (clinical: adverse outcomes)** | **Course academic total** | **Course completion** | **UKFPO EPM total** | **UKFPO EPM decile (quartiles until 2013)** | **UKFPO SJT** | **UKFPO total** | **FtP declarations at registration - health** | **FtP declarations at registration - conduct** |
| --- | --- | --- | --- | --- | --- | --- | --- | --- | --- | --- | --- | --- | --- | --- | --- | --- | --- |
| **Cognitive total** | **Tiffin et al., 2016** | **+ SS** | **+ SS** |  | **+ SS** | **+ SS** |  |  |  |  |  |  |  |  |  |  |  |
|  | **MacKenzie et al., 2016** |  |  |  |  |  |  |  |  |  |  | **+ SS** | **+ SS** | **+ SS** | **+ SS** |  |  |
|  | **McManus et al., 2013** | **+ SS** | No effect SS | **+ SS** |  |  |  |  |  |  |  |  |  |  |  |  |  |
|  | **Garrud et al., 2018 (univariate regression)** |  |  |  |  |  |  |  |  |  | **+? SS** |  | **+? SS** | +? SS |  |  |  |
|  | **Garrud et al., 2018 (simple correlation - graduates)** |  |  |  |  |  |  |  |  |  |  |  | **+ SS** | **++ SS** |  |  |  |
|  | **Mwandigha et al., 2018** | **+ SS** | No effect SS |  | **+ SS** | No effect SS |  |  |  |  |  |  |  |  |  |  |  |
|  | **Tiffin et al., 2017** | **+ SS** | **+ SS** |  |  |  |  |  |  |  |  |  |  |  |  |  |  |
|  | **Husbands et al., 2013** | **Mixed (+ve)** | **Mixed (+ve)** |  |  |  |  |  |  |  |  |  |  |  |  |  |  |
|  | **Lynch et al., 2013** | No effect NSS | No effect NSS |  |  |  |  |  |  |  |  |  |  |  |  |  |  |
|  | **Husbands et al., 2014** |  |  |  | **+ SS** | **Mixed (+ve)** |  |  |  |  |  |  |  |  |  |  |  |
|  | **Lambe, 2018** | **++ SS** |  |  |  |  |  | - NSS |  |  |  |  |  |  |  |  |  |
|  | **Sartania et al., 2014** | No effect SS |  |  | No effect SS | No effect NSS |  |  |  | No effect SS |  | No effect SS |  |  |  |  |  |
|  | **Yates et al., 2010** | **Mixed (+ve)** | No effect NSS |  |  |  |  |  |  |  |  |  |  |  |  |  |  |
|  | **Yates et al., 2013** |  |  | **+ SS** | **Mixed (+ve)** | **+ SS** |  |  |  |  |  |  |  |  |  |  |  |
|  | **Srikathirkamanathan et al., 2017** |  |  |  | **Mixed (+ve)** | **Mixed (+ve)** | **Mixed (+ve)** |  |  |  |  |  |  |  |  |  |  |
|  | **Adam et al., 2015** |  |  |  | **Mixed (+ve)** | **Mixed (+ve)** |  |  | **Mixed (-ve)** |  |  |  |  |  |  |  |  |
|  | **Adam et al., 2012** |  |  | **++ SS** |  |  |  | **Mixed (+ve)** |  |  |  |  |  |  |  |  |  |
|  | **Lala et al., 2013** | + NSS |  |  |  |  |  |  |  |  |  |  |  |  |  |  |  |
|  |  |  |  |  |  |  |  |  |  |  |  |  |  |  |  |  |  |
| **Cognitive + SJT total** | **Foley et al., 2015** |  |  |  |  |  |  |  |  | No effect SS |  |  |  |  |  |  |  |
|  | **ppppp et al., 2016** | Mixed (mixed) | Mixed (mixed) |  |  |  |  |  |  |  |  |  |  |  |  |  |  |
|  |  |  |  |  |  |  |  |  |  |  |  |  |  |  |  |  |  |
| **Cognitive+SJT total (percentile)** | **Foley et al., 2015** |  |  |  |  |  |  |  |  | **+ SS** |  |  |  |  |  |  |  |
|  |  |  |  |  |  |  |  |  |  |  |  |  |  |  |  |  |  |
| **AR** | **Paton et al., 2018** |  |  |  |  |  |  |  |  |  |  |  |  |  |  |  | No effect (OR) SS |
|  | **Tiffin et al., 2016** | No effect SS | No effect SS |  | No effect SS | No effect SS |  |  |  |  |  |  |  |  |  |  |  |
|  | **MacKenzie et al., 2016** |  |  |  |  |  |  |  |  |  |  | **+ SS** | No effect SS | **+ SS** | **+ SS** |  |  |
|  | **McManus et al., 2013** | No effect SS | No effect SS | No effect SS |  |  |  |  |  |  |  |  |  |  |  |  |  |
|  | **Tiffin et al., 2017** | No effect NSS | No effect NSS |  |  |  |  |  |  |  |  |  |  |  |  |  |  |
|  | **Lynch et al., 2013** | No effect NSS | No effect NSS |  |  |  |  |  |  |  |  |  |  |  |  |  |  |
|  | **Sartania et al., 2014** | No effect NSS |  |  | No effect NSS | No effect NSS |  |  |  | No effect NSS |  | No effect NSS |  |  |  |  |  |
|  | **Yates et al., 2010** | No effect NSS | No effect NSS |  |  |  |  |  |  |  |  |  |  |  |  |  |  |
|  | **Yates et al., 2013** |  |  | No effect NSS | No effect NSS | No effect NSS |  |  |  |  |  |  |  |  |  |  |  |
|  | **Srikathirkamanathan et al., 2017** |  |  |  | No effect NSS | No effect NSS | No effect NSS |  |  |  |  |  |  |  |  |  |  |
|  | **Adam et al., 2015** |  |  |  | **Mixed (+ve)** | **Mixed (+ve)** |  |  | **Mixed (-ve)** |  |  |  |  |  |  |  |  |
|  | **Adam et al., 2012** |  |  | **Mixed (+ve)** |  |  |  | **Mixed (+ve)** |  |  |  |  |  |  |  |  |  |
|  | **Lala et al., 2013** | Mixed (mixed) |  |  |  |  |  |  |  |  |  |  |  |  |  |  |  |
|  |  |  |  |  |  |  |  |  |  |  |  |  |  |  |  |  |  |
| **DA** | **Tiffin et al., 2016** | **+ SS** | No effect SS |  | **+ SS** | No effect SS |  |  |  |  |  |  |  |  |  |  |  |
|  | **MacKenzie et al., 2016** |  |  |  |  |  |  |  |  |  |  | **+ SS** | No effect SS | **+ SS** | **+ SS** |  |  |
|  | **McManus et al., 2013** | No effect SS | No effect SS | No effect SS |  |  |  |  |  |  |  |  |  |  |  |  |  |
|  | **Tiffin et al., 2017** | **+ SS** | **+ SS** |  |  |  |  |  |  |  |  |  |  |  |  |  |  |
|  | **Lynch et al., 2013** | No effect NSS | No effect NSS |  |  |  |  |  |  |  |  |  |  |  |  |  |  |
|  | **Sartania et al., 2014** | No effect SS |  |  | No effect NSS | No effect NSS |  |  |  | No effect SS |  | No effect NSS |  |  |  |  |  |
|  | **Yates et al., 2010** | No effect NSS | No effect  NSS |  |  |  |  |  |  |  |  |  |  |  |  |  |  |
|  | **Yates et al., 2013** |  |  | No effect NSS | No effect NSS | No effect NSS |  |  |  |  |  |  |  |  |  |  |  |
|  | **Srikathirkamanathan et al., 2017** |  |  |  | **Mixed (+ve)** | **Mixed (+ve)** | **Mixed (+ve)** |  |  |  |  |  |  |  |  |  |  |
|  | **Adam et al., 2015** |  |  |  |  |  |  |  |  |  |  |  |  |  |  |  |  |
|  | **Adam et al., 2012** |  |  | **+ SS** |  |  |  | **Mixed (+ve)** |  |  |  |  |  |  |  |  |  |
|  | **Lala et al., 2013** | **+ SS** |  |  |  |  |  |  |  |  |  |  |  |  |  |  |  |
|  |  |  |  |  |  |  |  |  |  |  |  |  |  |  |  |  |  |
| **QR** | **Tiffin et al., 2016** | No effect SS | No effect NSS |  | **+ SS** | No effect SS |  |  |  |  |  |  |  |  |  |  |  |
|  | **MacKenzie et al., 2016** |  |  |  |  |  |  |  |  |  |  | No effect SS | No effect SS | **+ SS** | **+ SS** |  |  |
|  | **McManus et al., 2013** | No effect SS | No effect SS | No effect SS |  |  |  |  |  |  |  |  |  |  |  |  |  |
|  | **Tiffin et al., 2017** | **+ SS** | No effect SS |  |  |  |  |  |  |  |  |  |  |  |  |  |  |
|  | **Lynch et al., 2013** | No effect NSS | No effect NSS |  |  |  |  |  |  |  |  |  |  |  |  |  |  |
|  | **Sartania et al., 2014** | No effect SS |  |  | No effect SS | No effect NSS |  |  |  | No effect SS |  | No effect SS |  |  |  |  |  |
|  | **Yates et al., 2010** | **Mixed (+ve)** | No effect NSS |  |  |  |  |  |  |  |  |  |  |  |  |  |  |
|  | **Yates et al., 2013** |  |  | **Mixed (+ve)** | No effect NSS | **Mixed (+ve)** |  |  |  |  |  |  |  |  |  |  |  |
|  | **Srikathirkamanathan et al., 2017** |  |  |  | **Mixed (+ve)** | No effect NSS | **Mixed (+ve)** |  |  |  |  |  |  |  |  |  |  |
|  | **Adam et al., 2015** |  |  |  |  |  |  |  | **Mixed (-ve)** |  |  |  |  |  |  |  |  |
|  | **Adam et al., 2012** |  |  | **Mixed (+ve)** |  |  |  | **Mixed (-ve)** |  |  |  |  |  |  |  |  |  |
|  | **Lala et al., 2013** | Mixed (mixed) |  |  |  |  |  |  |  |  |  |  |  |  |  |  |  |
|  |  |  |  |  |  |  |  |  |  |  |  |  |  |  |  |  |  |
| **VR** | **Paton et al., 2018** |  |  |  |  |  |  |  |  |  |  |  |  |  |  | No effect (OR) SS | No effect (OR) SS |
|  | **Tiffin et al., 2016** | **+ SS** | No effect SS |  | **+ SS** | **+ SS** |  |  |  |  |  |  |  |  |  |  |  |
|  | **MacKenzie et al., 2016** |  |  |  |  |  |  |  |  |  |  | **+ SS** | **+ SS** | **+ SS** | **+ SS** |  |  |
|  | **McManus et al., 2013** | **+ SS** | No effect NSS | **+ SS** |  |  |  |  |  |  |  |  |  |  |  |  |  |
|  | **Tiffin et al., 2017** | **+ SS** | No effect NSS |  |  |  |  |  |  |  |  |  |  |  |  |  |  |
|  | **Lynch et al., 2013** | + NSS | No effect NSS |  |  |  |  |  |  |  |  |  |  |  |  |  |  |
|  | **Sartania et al., 2014** | No effect SS |  |  | No effect SS | No effect NSS |  |  |  | No effect SS |  | No effect SS |  |  |  |  |  |
|  | **Yates et al., 2010** | **Mixed (+ve)** | No effect NSS |  |  |  |  |  |  |  |  |  |  |  |  |  |  |
|  | **Yates et al., 2013** |  |  | **+ SS** | **Mixed (+ve)** | **+ SS** |  |  |  |  |  |  |  |  |  |  |  |
|  | **Srikathirkamanathan et al., 2017** |  |  |  | **Mixed (+ve)** | **Mixed (+ve)** | **Mixed (+ve)** |  |  |  |  |  |  |  |  |  |  |
|  | **Adam et al., 2015** |  |  |  |  | **Mixed (+ve)** |  |  | **- SS** |  |  |  |  |  |  |  |  |
|  | **Adam et al., 2012** |  |  | **Mixed (+ve)** |  |  |  | **Mixed (-ve)** |  |  |  |  |  |  |  |  |  |
|  | **Lala et al., 2013** | Mixed (mixed) |  |  |  |  |  |  |  |  |  |  |  |  |  |  |  |
|  |  |  |  |  |  |  |  |  |  |  |  |  |  |  |  |  |  |
| **SJT** | **Tiffin et al., 2017** | **+ SS** | No effect SS |  |  |  |  |  |  |  |  |  |  |  |  |  |  |
|  | **Patterson et al., 2014** |  |  |  |  |  |  | **Mixed (+ve)** |  |  |  |  |  |  |  |  |  |
|  | **Lambe, 2018** | - NSS |  |  |  |  |  | - NSS |  |  |  |  |  |  |  |  |  |
|  | **Patterson et al., 2017** |  |  |  |  |  |  | **+ SS** |  |  |  |  |  |  |  |  |  |

Appendix Table 6: Univariate correlation results matrix. AR: Abstract Reasoning, DA: Decision Analysis, QR: Quantitative Reasoning, VR: Verbal Reasoning, FtP: Fitness to Practise, UKFPO: UK Foundation Programme, EPM: Educational Performance Measure, SJT: Situational Judgement Test, FtP: Fitness to practise, SS: Statistically Significant, NSS: Not Statistically Significant, OR: Odds Ratio.

| **UKCAT Subtest** | **Study** | **Pre-clinical knowledge-based** | **Pre-clinical skills-based** | **Pre-clinical total** | **Clinical knowledge-based** | **Clinical skills-based** | **Clinical total** | **Prof behaviour in MS (pre-clinical)** | **Professional behaviour in MS (clinical: adverse outcomes)** | **Course academic total** | **Course completion** | **UKFPO EPM total** | **UKFPO EPM decile (quartiles until 2013)** | **UKFPO SJT** | **UKFPO total** | **FtP declarations at registration - health** | **FtP declarations at registration - conduct** |
| --- | --- | --- | --- | --- | --- | --- | --- | --- | --- | --- | --- | --- | --- | --- | --- | --- | --- |
| **Cognitive total** | **Curtis et al., 2020** |  |  |  | **+ SS** |  |  |  |  |  |  | **+ SS** |  | No effect NSS |  |  |  |
|  | **Wright et al., 2010** |  |  |  |  |  | Mixed (mixed) |  |  |  |  |  |  |  |  |  |  |

Appendix Table 7: Multivariate correlation results matrix.
